# Intervening to change problematic alcohol use: using online personalized feedback to encourage changes in behaviour

**DOI:** 10.1186/1940-0640-8-S1-A11

**Published:** 2013-09-04

**Authors:** Bridgette M Bewick

**Affiliations:** 1 School of Medicine, Leeds Institute of Health Sciences, University of Leeds, UK

## 

This presentation outlines the development and evaluation of two online brief personalized feedback interventions for alcohol use (http://www.Unitcheck.co.uk and http://www.ChangeDrinking.com). By pulling together results from five individual studies, we aim to enable the audience to explore how the overall narrative enables additional insight in to our understanding of development, effectiveness, and implementation of online interventions. Methods and results from studies will be explained and contrasted. The theoretical underpinnings of the development of Unitcheck, an intervention initially developed for UK university students, will be explored briefly before outlining results of three randomised control trials. The results of a study employing qualitative think-aloud methodology will then be presented. Evidence will be presented of the ability of Unitcheck to prompt users to actively engage with information presented, relating information to their own experience; resulting in an evaluation of their own drinking behaviour. Evidence will be presented of user's immediate cognitive and emotional reaction when comparing their own drinking with that of others. Next, we discuss how experiences with Unitcheck were applied to inform development of ChangeDrinking, an online screening and brief advice intervention for non-help-seeking individuals who have recently been hospital inpatients. The results of a study employing qualitative think-aloud methodology to understand how those identified as having substance misuse problems engage with ChangeDrinking will then be presented. The presentation will consider how context of implementation (e.g. university vs national hospital) shaped development of each intervention tool and subsequently impacted the evaluation process. The synthesis of the results of the think-aloud studies with randomised control trial results enables insight in to potential mechanisms for behaviour change. This paper will consider the implication of results for our understanding of the use of online brief personalized feedback interventions for modifying alcohol use.

